# Therapeutic Effect of *Agaricus brasiliensis* on Phenylhydrazine-Induced Neonatal Jaundice in Rats

**DOI:** 10.1155/2015/651218

**Published:** 2015-03-25

**Authors:** Lan Zhang, Bo Yuan, HuiPing Wang, Ya Gao

**Affiliations:** ^1^Department of Pediatrics, Second Hospital of Xi'an Jiaotong University, Xi'an 710004, China; ^2^Xi'an Central Hospital, Xi'an 710003, China

## Abstract

The present study was designed to investigate the effect of *Agaricus brasiliensis* extract (ABE) on phenylhydrazine-induced neonatal jaundice in rats. Administration of ABE dose-dependently reduced the elevated bilirubin level induced by phenylhydrazine. It can be somewhat supported from the results of *in vitro* bilirubin degradation experiment. ABE treatment also reduced the total antioxidant status (TAOS), cascade O_2_^−^/SOD, level of NF-*κ*B protein, and adrenomedullin (AM). Overall, the results of this study demonstrated that *Agaricus brasiliensis* extract may be beneficial to reducing bilirubin level without causing hepatotoxicity in neonatal jaundice.

## 1. Introduction

Neonatal jaundice (NJ) occurs in more than 60% of normal newborns during their first week of life [1, 2]. The condition results from the abrupt cessation of bilirubin (BR) clearance by the placenta and transient deficiency in hepatic BR uptake, intracellular transport, and glucuronyltransferase conjugation [3]. It is a leading, yet preventable, cause of newborn rehospitalizations, deaths, and disabilities globally [4, 5]. Phototherapy (PT), which involves exposing a newborn's skin to electric lamp-generated blue light, is the standard treatment for removing excessive bilirubin, except in extreme cases when exchange transfusion becomes necessary [6]. Its efficacy is dependent on the color (wavelength) and intensity (irradiance) of the light emitted during phototherapy, the exposed body surface area, and the duration of exposure [7]. Unfortunately, PT may not be available in many countries because of the lack of devices and/or of reliable electrical power. And the conventionally used phototherapy may be increasing the risk of bilirubin-induced neurotoxicity [8, 9].

Presently, the plant kingdom is a wide field to search for natural effective hepatoprotective agent that has no side effects. More than 400 plants with glucose lowering effect are known [10]. Mushrooms and primarily basidiomycetous fungi are popular and valuable foods that are low in calories and high in minerals, essential amino acids, vitamins, and fibers [11]. Some of them produce substances with potential medical effects and are called medicinal mushrooms [12].* Agaricus brasiliensis* is native to Brazil and is widely grown in Japan and China because of its medicinal properties. It has traditionally been used for the prevention of a range of diseases, including hepatitis [13]. A few studies have researched that* A. brasiliensis* extract could ameliorate or abrogate CCL_4_-induced liver injury in rats [13, 14]. Hsu et al. [15] performed a 1-year open-label pilot study to observe whether* A. brasiliensis* extract improves liver function in patients with hepatitis B. However, the role of* A. brasiliensis* in neonatal jaundice has not been investigated. The aim of the present investigation was to evaluate the therapeutic effect of* Agaricus brasiliensis* in phenylhydrazine-induced neonatal jaundice rat model in order to propose a more effective and safer treatment for neonatal jaundice.

## 2. Materials and Methods

### 2.1. Animals

Healthy male adult Wistar rats (1 week old) were used in the study. The study was approved by Xi'an Jiaotong University Ethics Committee, and all procedures complied with the guidance set out in the Guidelines for Caring for Experimental Animals published by the Ministry of Science and Technology of the People's Republic of China. Every care was taken to minimize discomfort, distress, and pain.

### 2.2. *Agaricus brasiliensis* Extract (ABE)

The fermented mushroom of* Agaricus brasiliensis* was produced by the way introduced by Wang et al. [16]. The aqueous extraction was performed by adding 100 mL boiling water to 10 g air-dried mycelium. The infusion stood at room temperature for 30 minutes. After cooling and filtration, the extract was frozen and concentrated by lyophilization for five days overnight, in order to obtain the ABE (0.68 g).

### 2.3. Experimental Design

Animals were fasted for 12 h and were then injected (i.v.) with phenylhydrazine hydrochloride (75 mg/kg) solution that was made with physiological saline once daily for two consecutive days [17]. Forty NJ rats were selected and allocated equally into 4 groups. From then on, the 4 groups of rats of NJ were administered (i.g.) ABE 20 mg/kg/d, ABE 50 mg/kg/d, ABE 100 mg/kg/d, and saline, respectively. The other 10 normal rats were injected (i.v.) with the normal saline and used as the control group. All the treatments were done 4 h after phenylhydrazine administration on the second day. At the end of the experimental period (14 days later), the rats were fasted overnight and sacrificed by cervical dislocation. Blood samples drawn from the orbital sinus of the rats before killing were collected in plain and heparin vials for measuring the bilirubin level and liver function enzyme activity as well as O_2_^−^ and superoxide dismutase (SOD).

### 2.4. Measurement of Serum Bilirubin

The serum total bilirubin (STB), conjugated bilirubin (SCB), and unconjugated bilirubin (SUB) levels were measured using commercial kits (Shanghai Jinma Biological Technology, Inc., China) following the manufacturer's instructions.

### 2.5. Measurement of Serum ALT and AST

Serum ALT and AST activity were measured colorimetrically using a diagnostic kit (Shanghai Jinma Biological Technology, Inc., China) according to the instructions provided.

### 2.6. Histopathological Studies

Liver samples were collected and fixed in formalin for histology study. The formalin-fixed paraffin tissue sections were processed for staining with hematoxylin and eosin and then studied by light microscopy.

### 2.7. Measurement of Total Antioxidant Status

The total antioxidant status (TAOS) of liver was determined as previously described by Han [18]. The increase of absorbance at 405 nm was measured by a microplate reader (Shanghai Xunda Medical Technology, Inc., China).

### 2.8. Measurement of Cascade O_2_^−^/SOD

The level of O_2_^−^ was measured by NBT (nitro blue tetrazolium) reaction in TRIS buffer in the plasma and spectrophotometrically read at 530 nm. The activity of SOD was measured according to the method of Misra and Fridovich [19]. Catalase activity was measured by the method of Beutler [20].

### 2.9. Measurement of NF-*κ*B Activity

We used 10 *μ*g of the nuclear extract from each liver. Activated NF-*κ*B was quantified in liver tissue extracts via ELISA technique using the PathScan Phospho-NF*κ*B p65 (Ser536) Sandwich ELISA Antibody Pair (Shanghai Xunda Medical Technology, Inc., China), following the manufacturer's instructions. The protein expression levels of NF-*κ*B were measured by Western blot analysis.

### 2.10. Measurement of AM Levels

Serum AM levels were measured with ELISA method (Shanghai Xunda Medical Technology, Inc., China). Results were given as ng/mL.

### 2.11. *In Vitro* Bilirubin Degradation Experiment

Bilirubin (Sigma Chemical Co., Shanghai, China) was dissolved in a buffer solution (18.5% 0.1 N NaOH, 44.5% human albumin, and 37% 0.055 M Na_2_HPO_4_), with the final concentration and pH adjusted to 20 mg/dL and 7.4, respectively. In each group, ten microhematocrit tubes containing 100 L of the bilirubin solution per tube with or without different concentrations of ABE were for 5 h at room temperature. Bilirubin concentration was measured with Bilirubin Stat-Analyzer (Advanced Instruments Inc., USA).

### 2.12. Statistical Analysis

The data were analyzed using SPSS and expressed as means ± standard deviation (SD). Differences were considered statistically significant when *P* < 0.05 by one-way analysis of variance (ANOVA).

## 3. Results and Discussions

**Figure 1:**
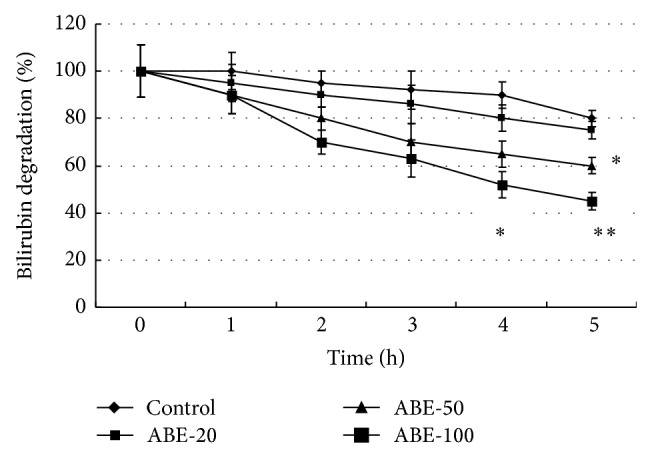
Comparison of* in vitro* efficacy of bilirubin degradation between different concentrations of ABE. Values are shown as means ± SEM. ^*^ *P* < 0.05 versus control group, ^**^ *P* < 0.01 versus control group.

Neonatal jaundice occurs in newborns as a result of excessive bilirubin formation and transient inability of the neonatal liver to clear bilirubin rapidly enough from the blood. Severe hyperbilirubinemia is toxic to the developing central nervous system [21]. Prolonged and uncontrolled high levels of bilirubin lead to bilirubin encephalopathy and subsequently kernicterus [22]. We used phenylhydrazine to induce nonhepatic neonatal hyperbilirubinemia in rats because it increased unconjugated bilirubin level. The bilirubin level was measured to evaluate the role of ABE in NJ. The serum STB levels in control group were found to be 0.4 ± 0.07 mg/dL. A significant increase in the serum STB in serum was observed in phenylhydrazine group, as compared to the control group (*P* < 0.01), whereas ABE decreased the STB and SUB levels significantly in a dose-dependent manner (Table 1). It can be somewhat supported from the results of* in vitro* bilirubin degradation experiment (Figure 1). Figure 1 shows the action of the ABE in bilirubin solution. Treatment of ABE shows a significantly higher efficacy of bilirubin gradation than the control group in* in vitro* experiment in a dose dependent manner. This result indicated that the effects of ABE on bilirubin gradation may be one of its important mechanisms in fighting neonatal jaundice.

**Figure 2:**
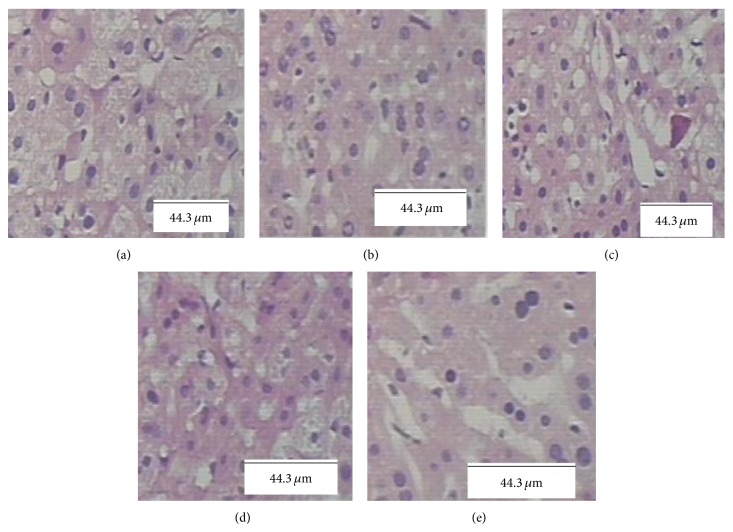
Histopathological analysis of rat liver sections using hematoxylin and eosin staining. (a) Section from a normal control rat liver. (b) Section from ABE-100 rat liver. (c) Section from ABE-50 rat liver. (d) Section from ABE-20 rat liver. (e) Section from a phenylhydrazine rat liver.

Phenylhydrazine induces neonatal jaundice conditions because it increases unconjugated bilirubin level without any significant change in the liver function. Liver function was evaluated by assessing serum ALT and AST, since AST and ALT are sensitive indicators of liver cell injury [23]. In the present study, we have confirmed that phenylhydrazine did not increase AST and ALT levels in serum of rats (Table 2). Consistent with the result, the liver histopathological observations also did not show any hepatic damage due to phenylhydrazine administration. As hypothesized, normal activity of the liver function enzymes and absence of any liver damage after ABE administration indicated the safety profile of ABE (Figure 2).

**Figure 3:**
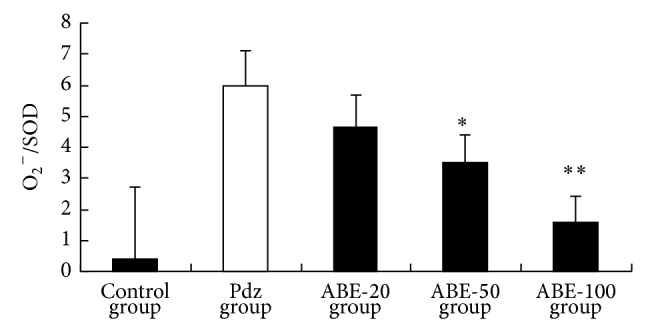
Effect of ABE on cascade of O_2_^−^/SOD values. Values are shown as means ± SEM. ^*^ *P* < 0.05 versus phenylhydrazine, ^**^ *P* < 0.01 versus phenylhydrazine group (Pdz: phenylhydrazine).

Several studies have shown that the antioxidant defense system is altered during pregnancy. Exposure to oxidative stress may result in excessive bilirubin production that, when combined with diminished conjugation capacity, severely exacerbates the potential for extreme hyperbilirubinemia [24]. Neonatal erythrocytes are prone to oxidative damage due to their unsaturated membrane lipids [25]. In this study, phenylhydrazine administration increased TAOS activity (Table 3). We measured TAOS activity as an indirect indication of the formation of O_2_^−^ and other oxidant species. Those in the ABE-50- and ABE-100-treated groups were significantly lower than those in the phenylhydrazine-treated group (*P* < 0.05 and *P* < 0.01, resp.). Moreover, significantly higher cascade of O_2_^−^/SOD values was measured in the phenylhydrazine group (*P* < 0.01) compared to the control group. ABE-100 suppressed phenylhydrazine and induced the higher cascade of O_2_^−^/SOD values. Taken together, these results clearly indicated that oxidative stress was generated in erythrocyte of neonatal jaundiced mice. ABE treatment reversed these adverse effects (Figure 3).

**Figure 4:**
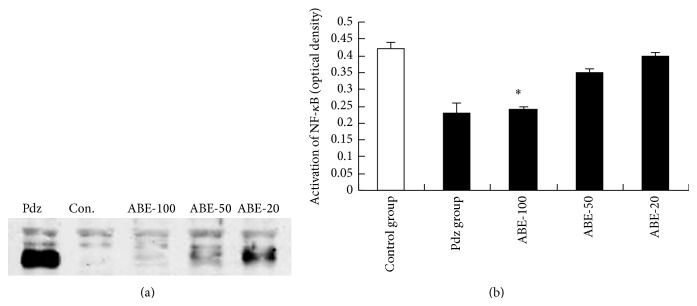
Effect of ABE on protein expression of NF-*κ*B. Values represent the mean ± SEM. ^*^ *P* < 0.05 versus phenylhydrazine group (Pdz: phenylhydrazine).

An extreme high level of SUB elicits the release of proinflammatory cytokines, such as TNF-*α* and IL-1*β*, through the activation of NF-*κ*B signaling pathways at the intracellular level [26, 27]. Substantially, hyperbilirubinemia induced a predominant increase in nuclear translocation of NF-*κ*B (Figure 4(a)). As expected, level of NF-*κ*B protein decreased in the nucleus of liver cells of ABE-100 group (Figure 4(b)). Data obtained from the treatment of rats with phenylhydrazine indicated that NF-*κ*B played an important role in hyperbilirubinemia. The increased NF-*κ*B protein expression was attenuated by ABE-100. It indicated that ABE performs its neonatal jaundice protective effect, at least partly, due to the regulation on NF-*κ*B, which can further influence the release of proinflammatory cytokines.

**Figure 5:**
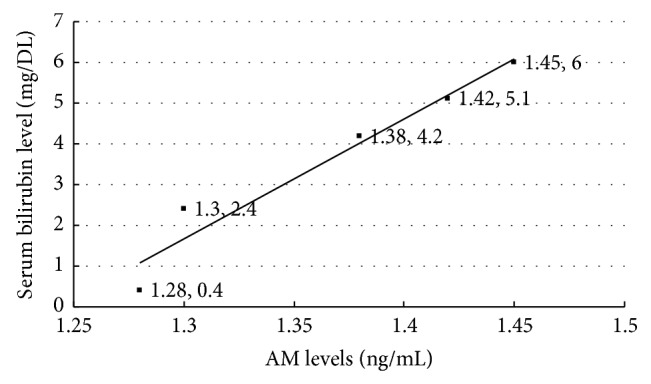
Correlation between serum AM levels and serum bilirubin levels.

Adrenomedullin (AM) is a peptide with 52 amino acids and has tyrosine amino acid at carboxy terminal. It plays a significant role in adverse effects and neuronal injury steps of significant hyperbilirubinemia [28]. In this study, there were statistically significant differences between the phenylhydrazine and control groups regarding AM levels (*P* < 0.001). Compared to phenylhydrazine group, ABE administration reduced AM levels in a dose-dependent manner (Table 4). In further analysis, there was a significant positive correlation between serum bilirubin levels and simultaneously measured serum AM levels (Figure 5).

In conclusion, our findings showed that* Agaricus brasiliensis* extract prevented the progression of phenylhydrazine-induced neonatal jaundice in rats. The therapeutic mechanism of* Agaricus brasiliensis* extract not only included efficient bilirubin clearance potential but also reduced oxidative stress and regulation on NF-*κ*B without causing hepatotoxicity.* In vitro* bilirubin degradation experiment showed that effects of ABE on bilirubin gradation may be one of its important mechanisms in fighting neonatal jaundice. From this study, we could conclude that* Agaricus brasiliensis* extract may be used as a protective food or medicine for neonatal jaundice. The potential application of* Agaricus brasiliensis* extract needs to be further studied.

## Figures and Tables

**Table 1:** Effect of ABE on serum bilirubin level.

Different groups	STB (mg/DL)	SUB (mg/DL)	SCB (mg/DL)
Control group	0.4 ± 0.07^∗∗^	0.14 ± 0.03^∗∗^	0.33 ± 0.05^∗∗^

ABE-100	2.4 ± 0.50^∗∗^	1.4 ± 0.51^∗∗^	0.8 ± 0.50

ABE-50	4.2 ± 0.50^∗^	3.2 ± 0.50^∗^	0.6 ± 0.50
ABE-20	5.1 ± 0.60	4.1 ± 0.60	0.88 ± 0.60
Phenylhydrazine	6.0 ± 0.70	5.1 ± 0.70	0.6 ± 0.71

Values are shown as means ± SEM. ^*^ *P* < 0.05 versus phenylhydrazine; ^**^ *P* < 0.01 versus phenylhydrazine group.

**Table 2:** Effect of ABE on ALT and AST.

Different groups	ALT (U/L)	AST (U/L)
Control group	31.0 ± 3.2	36.0 ± 6.2
ABE-100	30.2 ± 1.0	31.5 ± 1.3
ABE-50	40.2 ± 1.5	34.5 ± 1.5
ABE-20	35.1 ± 8.0	32.6 ± 8.1
Phenylhydrazine	40.2 ± 1.0	40.5 ± 1.3

**Table 3:** Effect of ABE on TAOS activity (*µ*M L-ascorbate).

Different groups	TAOS activity (*µ*M L-ascorbate)
Control group	28.41 ± 3.17
ABE-100	48.35 ± 3.33^∗∗^
ABE-50	56.30 ± 4.00^∗^
ABE-20	66.22 ± 2.11
Phenylhydrazine	80.33 ± 9.32

Values are shown as means ± SEM. ^*^ *P* < 0.05 versus phenylhydrazine; ^**^ *P* < 0.01 versus phenylhydrazine group.

**Table 4:** Effect of ABE on AM levels (ng/mL).

Different groups	AM levels

Control group	1.28 ± 0.07
ABE-100	1.30 ± 0.03^∗∗∗^
ABE-50	1.38 ± 0.04^∗^
ABE-20	1.42 ± 0.11
Phenylhydrazine	1.45 ± 0.06

Values are shown as means ± SEM. ^*^ *P* < 0.05 versus phenylhydrazine; ^***^ *P* < 0.001 versus phenylhydrazine group.
